# Is Cutaneous T-Cell Lymphoma Caused by Ultraviolet Radiation? A Comparison of UV Mutational Signatures in Malignant Melanoma and Mycosis Fungoides

**DOI:** 10.3390/cells12121616

**Published:** 2023-06-13

**Authors:** Robert Gniadecki, Sandra O’Keefe, Dylan Hennessey, Aishwarya Iyer

**Affiliations:** Division of Dermatology, University of Alberta, Edmonton, AB T6G 2G3, Canada; sokeefe@ualberta.ca (S.O.); dhennes@ualberta.ca (D.H.); aiyer2@ualberta.ca (A.I.)

**Keywords:** cutaneous T-cell lymphoma, ultraviolet, mutation signatures, malignant melanoma, whole-exome sequencing

## Abstract

Ultraviolet (UV) radiation is a strong environmental carcinogen responsible for the pathogenesis of most skin cancers, including malignant melanoma (MM) and non-melanoma (keratinocyte) skin cancers. The carcinogenic role of UV was firmly established based on epidemiological evidence and molecular findings of the characteristic mutation signatures which occur during the excision repair of cyclobutane pyrimidine dimers and 6,4-photoproducts. The role of UV in the pathogenesis of mycosis fungoides (MF), the most common type of primary cutaneous T-cell lymphoma, remains controversial. Here, we performed whole-exome sequencing of 61 samples of MF cells microdissected from cutaneous lesions, and compared their mutational signatures to 340 MMs. The vast majority of MM mutations had a typical UV mutational signature (SBS 7, SBS 38, or DSB 1), underscoring the key role of ultraviolet as a mutagen. In contrast, the SBS 7 signature in MF comprised < 5% of all mutations. SBS 7 was higher in the intraepidermal MF cells (when compared to the dermal cells) and in the cells from tumors as compared to that in early-stage plaques. In conclusion, our data do not support the pathogenic role of UV in the pathogenesis of MF and suggest that the UV mutations are the result of the cumulative environmental ultraviolet exposure of cutaneous lesions rather than an early mutagenic event.

## 1. Introduction

Primary cutaneous lymphomas are lymphoid cancers that develop in the skin, and in most cases, run a relatively indolent course and rarely metastasize to the parenchymatous organs. Mycosis fungoides (MF) is the most common type of primary cutaneous T-cell lymphoma, manifesting itself clinically as scaly patches and plaques, and occasionally progressing to tumors [1]. The origin of MF has been a subject of considerable interest and remains controversial. The prevalent view is that MF represents a clonal expansion of a mature tissue-resident memory T-cell [2]. However, there is a considerable body of evidence that MF, such as many other lymphomas, develops from immature T-cell progenitors which differentiate into skin-homing, pre-malignant T-cells that colonize the skin, proliferate, and form MF lesions [3,4].

UV radiation is the most common environmental carcinogen implicated in the pathogenesis of most skin cancers. UV is mutagenic and plays a role in cancer initiation and promotion [5]. The risk of malignant melanoma (MM) and non-melanoma (keratinocyte) skin cancers is strongly correlated with solar exposure and sunburns, and cancer cells show a high number of genomic UV-induced mutations [6,7,8]. A similar dependency on UV exposure would strongly support the origin of MF in the skin rather than from migratory immature T-cell precursors. However, the evidence linking MF to UV exposure has been inconclusive. Some authors found an epidemiological association between lymphoma risk and solar exposure [9,10,11], but those correlations were weak and were not reproduced by other investigators [12]. Other studies examined the patterns of mutations and reported putative UV signatures in specific oncogenes (TP53) and in the genomes in MF [13,14,15]. However, those studies examined crude skin biopsies which might have contaminated the material with epidermal cells harboring UV mutations. They also did not take into account the possible iatrogenic impact of phototherapy, which is a standard treatment for MF and is administered cyclically over extended periods of time.

Confirmation of a large number of UV mutations in MF genomes would indicate the causative role of solar radiation in its pathogenesis and favor the role of long-lived skin-resident T-cells rather than migratory immature precursors as the cells of origin. We therefore re-examined the question of whether UV mutational signatures are found in MF in this study. To avoid the biases identified in other studies, we carefully purified lymphoid tumor cells by microdissecting skin biopsies, analyzed the data using validated bioinformatic pipelines [16,17], and compared the MF data to the mutation patterns of malignant melanoma, which is the well-established example of a UV-dependent tumor [8].

## 2. Materials and Methods

### 2.1. Data Sources

For analysis of MF mutations, we used whole-exome sequencing data which were generated previously via microdissected biopsies from the lesional skin of patients with a confirmed diagnosis of mycosis fungoides (Table 1) [3,18].

Briefly, tumor cell clusters were prepared using laser-capture microdissection of the skin biopsies, and DNA was isolated from the microdissected materials pooled for each biopsy as well as from matched peripheral blood mononuclear cells and buccal swabs. Whole-exome sequencing (WES) libraries were prepared with the NEBNext Ultra II DNA Library Prep Kit for Illumina (New England Biolabs, Ipswich, MA, USA), and the exome and untranslated region DNA reads were captured using SSELXT Human All Exon V6 +UTR probes (Agilent Technologies, Santa Clara, CA, USA) and sequenced using Illumina (San Diego, CA, USA) HiSeq 1500 and Novaseq 6000 sequencers. Ethical approval HREBA.CC-16-0820-REN1 was obtained from the Health Research Ethics Board of the Alberta Cancer Committee. Whole-exome sequencing data that represented samples of malignant melanoma data were downloaded from The Cancer Genome Atlas (TCGA) (Table 2).

### 2.2. Data Analysis

The raw fastq files generated from WES were processed through the GATK (version 4.0.10) best practices workflow and aligned to the hg38 reference genome. Somatic variants (SVs), including single somatic mutations and indels, were identified by the MuTect2 variant caller (version 2.1). The synonymous and non-synonymous SVs were identified by the Variant Effect Predictor (VEP) (version 95.2). The VEP files were then used for identifying the single-base substitution (SBS) and insertion and deletion (ID) signatures using SignatureAnalyzer (version 0.0.8) [19]. Visual data representations were created using Prism (GraphPad software, version 9).

## 3. Results

### 3.1. UV Mutational Signature Is Dominant in Malignant Melanoma

**Figure 1:**
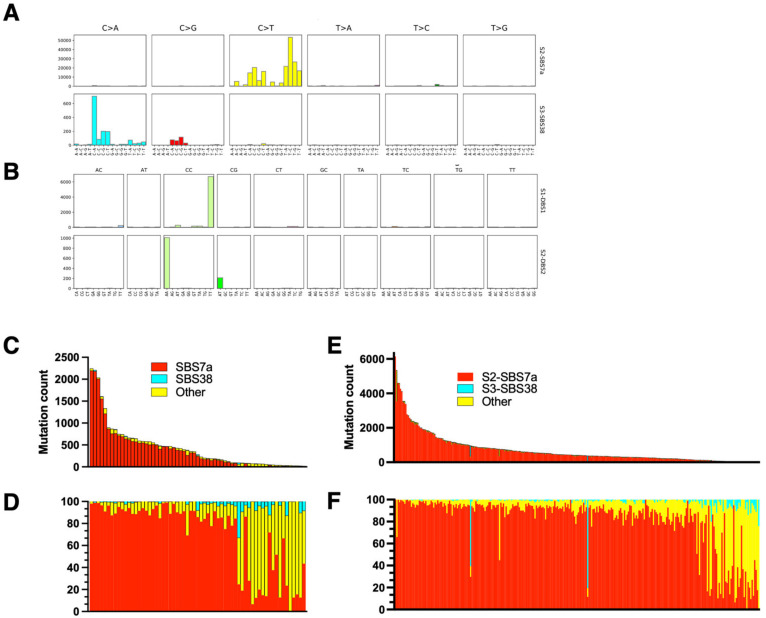
Mutation signatures of malignant melanoma. The exome sequences from primary cutaneous melanoma lesions and melanoma metastases were retrieved from The Cancer Genome Atlas (TCGA) and analyzed using SignatureAnalyzer, as described in the methods. The frequencies of single-base substitutions (SBS) and double-base substitutions (DBS) are shown in (**A**,**B**), respectively. (**C**–**F**) The absolute numbers of mutations per exome (**C**,**E**) and the relative frequencies of mutations (**D**,**F**) classified as UV signatures (SBS7a (red), SBS38 (cyan)) (shown as a percentage of all mutations). (**C**,**D**) Primary cutaneous melanoma (skin lesions); (**E**,**F**) metastatic melanoma (lymph nodes and organ metastases).

The UV signature of MM was investigated previously [8], and we performed an independent analysis of the TCGA dataset as a quality control of our analytic pipelines and as a reference for the MF samples. For the single-base substitution (SBS) UV mutations we detected SBS 7a and SBS 38 (Figure 1). The UV signature 7a comprises C>T substitutions at TpC that are probably formed during the repair of 6,4-photoproducts, and was the dominant signature in melanoma across all the samples. SBS 38 results from indirect DNA damage by UV and is characterized by a high proportion of C>A substitutions [8,16]. Interestingly, we did not detect other subtypes of SBS 7, i.e., SBS7b (C>T substitutions at CpC, reflecting the repair of cyclobutane pyrimidine dimers), or SBS 7c (a high proportion of T>A and T>C substitutions, such as SBS 38, reflecting indirect DNA damage), which were detected in smaller amounts relative to SBS7a in the previous benchmark study [8]. However, SBS 7a and SBS 7b signatures are very similar and may be misattributed to each other, especially when analyzing WES datasets which are noisier and resolve fewer mutations than obtainedfrom the whole-genome sequencing of Hayward et al. [8]. Importantly, we found an expected double-base substitution, DSB 1 (CC>TT), which is also a specific UV signature, albeit it is present in small quantities (<5% of SBS7a) [6,16,17,20]. In our material, DBS 1 and the less abundant DBS 2 (sometimes attributed to tobacco smoking) were the only DBS signatures and were present in 24 samples (Figure 1). There was a wide variation in the proportions and numbers of UV mutations across the samples, but on average, 92% of all mutations could be attributed to UV (SBS 7a + SBS 38 + DBS 1).

**Figure 2:**
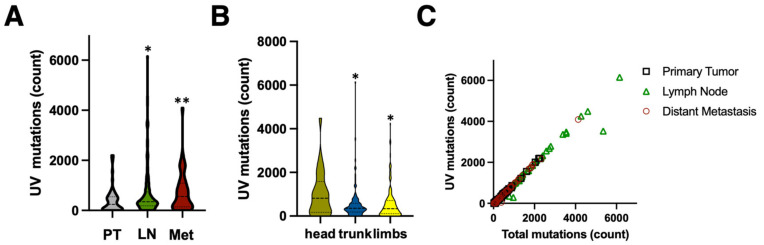
Properties of UV mutations in malignant melanoma. The UV mutations (SBS7 + SBS38) were called as in Figure 1. (**A**) Number of mutations in primary skin melanoma lesions (PT), melanoma lymph node metastases (LN), and in organ metastases (Met). Statistically significant difference for pairwise comparisons to PT: * *p* = 0.0092, ** *p* = 0.0029, unpaired Mann–Whitney. Panel (**B**) shows the number of mutations in melanoma skin lesions in different regions of the body. Statistically significant difference for pairwise comparisons to ‘head’: * *p* = 0.02, unpaired Mann–Whitney. (**C**) Correlation between total number of mutations and UV mutations.

We compared UV mutations in samples of cutaneous melanoma to the samples obtained from lymph nodes and distal metastases (Figure 1C,E and Figure 2A). As expected, the skin lesions had a lower UV mutation burden compared to lymph node and distal metastases (mean 395 (95% CI 268–522) vs. 674 (CI 534–814) vs. 738 (CI 490–987), *p* = 0.01 for pairwise comparisons, Mann–Whitney test). Melanomas on the sun-exposed skin on the head had a higher UV mutation burden than melanomas on the trunk and limbs (1021 (CI 212–1063) vs. 552 (CI 44–731), *p* = 0.02, Mann–Whitney test) (Figure 2B). There was no difference between the UV mutation burden of T3 and T4 stage cutaneous melanomas (not shown), but there was a good correlation between the total mutation burden and number of UV mutations (SBS 7a + SBS 38 + DBS 1), as reported previously [7,8] (Figure 2C).

### 3.2. UV Mutations in Mycosis Fungoides

**Figure 3:**
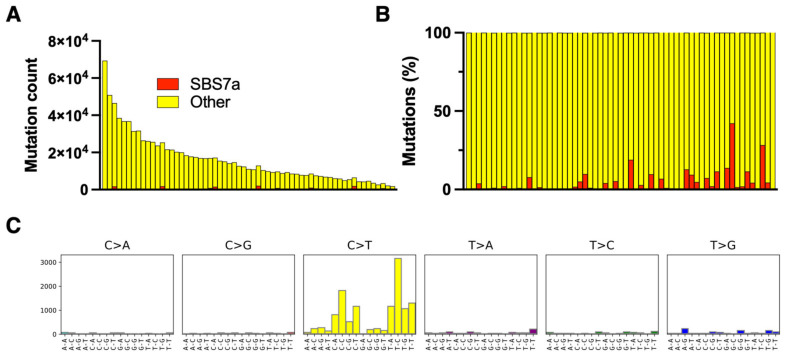
Mutation signatures in mycosis fungoides (MF). The clusters of malignant cells were microdissected via whole-exome sequencing. Mutation signatures were obtained using the pipelines for malignant melanoma (Figure 1). The absolute numbers of mutations per exome (**A**) and the relative frequencies of mutations (**B**) classified as UV signatures (SBS7a). (**C**) The SBS signature of SBS7a in MF.

All samples included in the study were microdissected clusters of tumor cells, and therefore, they had a high tumor cell fraction and were not contaminated with epithelial tissue, which is a rich source of mutated DNA [3,18]. MF had a high total mutation burden comparable to MM, but the only UV-related mutation (SBS 7a) only comprised 4% (CI 2–6%) of the mutation burden (Figure 3).

**Figure 4:**
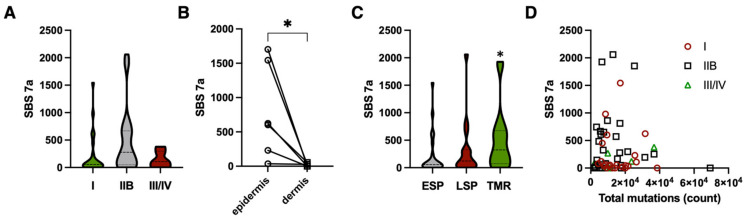
Characteristics of UV mutations in MF. The UV mutations (SBS7 + SBS38) were called as in Figure 3. (**A**) Number of mutations in primary skin MF lesions from patients with stages I, IIB, and advanced stages (III and higher). There was a trend towards a lower number of mutations in stage I vs. IIB and III/IV (*p* = 0.034, Brown-Forsythe and Welch ANOVA test for samples with unequal standard deviations). (**B**) Number of mutations in malignant lymphocytes from the epidermis and from the deeper dermal infiltrates within the same lesion. * *p* = 0.031, Wilcoxon matched-pairs rank test. (**C**) Number of UV mutations depending on the type of lesion (ESP—early-stage plaque (stages I-IIA), LSP—late-stage plaque (plaque lesions in patients with stage IIB and higher) and in tumors([stage IIB or higher). There was no difference between ESP and LSP, but TMR had a significantly higher number of mutations (* *p* = 0.04). (**D**) Lack of correlation between total number of mutations and UV mutations.

The low UV mutation burden indicated that they are unlikely to occur early in MF development, but were rather passively accumulated due to exposure of the lesions to UV (from the sun and during phototherapy). We have therefore hypothesized that long-standing lesions will have a higher number of UV mutations than the lesions in the early stage of the disease. As the duration of the lesions are roughly correlated with the stage of the disease, we compared samples from early-stage patients (stage IA and IB) to advanced-stage patients (IIB-IV). As predicted, stage I disease had a lower number of UV mutations than stage IIB (231 (CI 57–404) vs. 486 (CI 250–721), *p* = 0.05 Mann–Whitney test, significant rend of *p* = 0.034, Brown-Forsythe and Welch ANOVA test for samples with unequal standard deviations). The UV mutations were not correlated to the total mutation burden (Figure 4A,B).

Interestingly, lesions from stage III (erythrodermic patients) and IV (distal metastases) had a lower UV mutation burden, which might be caused by the rapid evolution and growth of the skin lesions in those stages and the dilution of the accumulated UV mutations. To further investigate this, we divided our samples into three categories according to their morphology and the stage of the disease [18,21,22,23]. The early-stage plaques (ESPs) are found in patients with early-stage disease (stage IA/IB). Samples from patients with advanced MF (stage ≥ IIB) were classified based on their morphology as tumors (TMRs) or late-stage plaques (LSPs). LSPs morphologically resemble the lesions in early-stage disease and are sometimes carried over from early MF where they develop de novo in patients already in a stage ≥ IIB. A comparison of the UV mutation load between these lesions revealed a trend towards a higher UV burden in the more advanced, long-standing lesions (ESP = 231 (CI 57–404); LSP = 327 (CI 139–538); TMR = 504 (135–572), *p* = 0.04 (Mann–Whitney) for the difference between the ESP and TMR) (Figure 4C).

Finally, if the UV mutations were mainly caused by the cumulative exposure of the established lesions to UV, we hypothesized that the tumor cells in the more superficial layers of the skin (epidermis) would have a higher UV mutation load than the cells in the dermis. We were able to microdissect intraepidermal malignant lymphocytes (Pautier abscesses) from the dermal malignant cells in 6 samples and compared their SBS 7a signatures (Figure 4D). As was predicted, the epidermal malignant lymphocytes had a strikingly higher UV mutation burden than dermal cells in the same biopsies.

## 4. Discussion

Ultraviolet is a well-characterized environmental mutagen and a complete carcinogen, playing a prominent role in the pathogenesis of skin cancers. Common skin cancers, such as malignant melanoma, basal cell carcinoma, and squamous cell carcinoma most likely originate from immature melanocytic and keratinocyte stem cells, residing in the bulge area of the hair follicle and also in the interfollicular epidermis [24,25,26,27,28,29]. These cells are non-migratory and slowly proliferate, remaining in the same anatomical niche for decades where they are exposed to environmental UV radiation and slowly accumulate mutations that eventually lead to malignant transformation. Therefore, the UV mutation load is high in early skin cancers and resembles the mutation load in non-neoplastic, UV-damaged skin [30,31].

Thus, a high percentage of UV mutations is a hallmark of all skin cancers where UV plays a decisive pathogenic role. Melanomas which develop in sun-protected areas, such as the mucosa or the uveal melanoma, are not dependent on UV as a carcinogen, and their UV signature mutations are absent or present in small quantities [32,33]. In our own analysis, >90% of all malignant melanoma mutations had a UV signature of SBS 7a, with a smaller component of SBS 38 and DBS 1. We also confirmed that the UV signature correlated with the expected level of exposure to UV, and tended to be higher for MM localized in the face versus the trunk and limbs [7]. Interestingly, those signatures persisted in metastatic tumors in the lymph nodes and in parenchymal organs without ongoing UV exposure. Similar findings have been reported before, and it was even suggested that UV mutations can be used to determine whether organ metastases without clinically identifiable primary melanoma arise from occult primary cutaneous lesions [34]. Thus, analyses of the UV mutation signatures confirmed the pattern characteristic for all UV-dependent cancers: a high percentage of UV mutations which persist during tumor evolution and which are correlated with the degree of exposure of the skin region to solar radiation.

It has been a matter of debate whether UV plays a role in the development of cutaneous lymphomas, such as mycosis fungoides (MF). Epidemiological evidence has not been convincing, and MF tends to develop primarily in sun-protected areas (buttocks and inner thighs) unlike UV-dependent cancers that have a predilection to the areas exposed to UV or that have suffered previous sun burns. Moreover, the migratory nature of the lymphocytes argues against the role of UV because these cells are unlikely to dwell in the skin long enough to accumulate UV mutations. However, the discovery and characterization of skin-resident T-cells rekindled the debate about the relevance of UV as a carcinogen in MF. The resident memory T-cells do not readily recirculate but persist in the skin, and are therefore exposed to UV to the same degree as native skin cells such as keratinocytes or melanocytes [35,36]. These skin-resident T-cells were proposed to be the cell of origin for MF [2], and C>T mutations were found in MF (including the TP53 gene [13,15]), further supporting the importance of UV [14].

The results of our analysis of UV mutation signatures in MF argue against the role of ultraviolet in the pathogenesis of this lymphoma. The UV signature mutations (SBS 7a) are found in small quantities and their contribution to the total mutational load is negligible (≤5%), which is a striking contrast to that of UV-dependent tumors such as melanoma. This small proportion of UV mutations could easily be explained as a result of the normal solar exposure of MF lesions, and they persist as passenger mutations. Since patients with MF are usually treated with phototherapy, therapeutic exposure to UV would further contribute to UV mutations.

The fact that UV signature mutations are more frequent in the epidermal lymphocytes as compared to the lymphocytes in the dermis further supports this interpretation. The carcinogenic UVB radiation penetrates to the level of the papillary dermis, but its energy is rapidly dissipated by the stratum corneum (scattering and reflection of UV) and by the epidermal melanin that provides 20–50% of the photoprotection [37,38]. Thus, both epidermal and superficial dermal lymphomatous infiltrates are exposed to UV, but the exposure levels are likely to be significantly higher in the epidermal lymphocytic abscesses, which are not efficiently photoprotected by epidermal melanin.

We also found that the amount of UV mutations increases slightly during the progression of the disease, most likely due to cumulative effects of sun exposure and ongoing phototherapy. Unfortunately, we were unable to determine the duration of the biopsied lesions, which would make the analysis more reliable.

The discrepancy between our findings and the results of other groups reporting significant UV signatures in MF [14] may be explained by the methods of sample preparation and analysis. Most sequencing studies in MF use entire skin biopsies. We have shown that lymphoma cells contribute to the minority of cells in bulk biopsies (usually less than 20% in stage I disease), with the rest being reactive lymphocytes, macrophages, and normal skin cells such as fibroblasts and epidermal cells. Considering the high UV mutation level in normal epidermis [31], even small amounts of keratinocytes would significantly increase the UV signature in the sample. We avoided this pitfall by carefully microdissecting malignant lymphoid cells and enriching the tumor cell fraction from the median 19.3% in crude biopsies to 69.3% (range of 21–98%) [4].

One of the limitations of our paper is the use of whole-exome sequencing data rather than using whole-genome sequencing. WES captures only the protein coding that comprises 1–2% of the genome, whereas whole-genome sequencing covers 99% of the genome. Thus, the number of mutations per genome available for analysis is much lower, which may impact the results. Moreover, UV mutagenesis is not a random event but depends on chromatin methylation. It was shown that UV-induced cyclobutane pyrimidine dimer DNA lesions are reduced within the demethylated CpG areas of gene promoters, which results in a unique trinucleotide UV signature with reduced TCG>TTG transitions [39]. In this study, we were unable to analyze such local variations in the UV signatures. Finally, to obtain better insight into the kinetics of UV damage in lymphoma cells, it would be desirable to compare the levels of mutations i the epidermis to that in the same area as the tumor. Epidermal UV mutations can serve as a molecular UV dosimeter and would allow for the capture of regional, cumulative UV mutagenesis.

## 5. Conclusions

Mutations caused by ultraviolet radiation are unlikely to cause cutaneous T-cell lymphoma (MF). The specific UV mutational signature (SBS 7a) has a very low frequency and most probably represents passenger mutations induced by environmental and therapeutic ultraviolet exposure. Our data support the previous epidemiological evidence which did not show correlations between UV and the risk of lymphoma, and may inform clinicians regarding the advice given to the patients about sun protection and the risk of phototherapy.

## Figures and Tables

**Table 1:** Characteristics of MF samples.

	All	ESP ^1^	LSP ^1^	TMR ^1^
Patients (number)	35			
Female (%)	25.70%			
White (%)	96%			
Age (mean [range])	68 (47–90)			
Samples (n)	61	27	14	20
IA	6	6	NA	NA
IB	21	21	NA	NA
IIB	28	NA	11	17
III	2	NA	1	1
IVA	4	NA	2	2

^1^ ESP—early-stage plaque, LSP—late-stage plaque, TMR—tumor, NA—not applicable.

**Table 2:** Characteristics of melanoma samples.

	Primary Tumor	Metastases			N/A	Total
		Regional ^1^	Lymph Node	Distant		
Patients (number)	63	61	170	43	3	340
Female (%)	36.5	37.7	37.1	39.5	33.3	37.4
White (%)	93.7	98.4	98.8	95.3	100.0	97.4
Age (mean [range])	63 (24–90)	57 (19–81)	54 (15–87)	58 (25–85)	74 (68–83)	61 (15–90)
Stage 1 (%)	0.0	16.4	27.1	16.3	0.0	18.5
Stage 2 (%)	54.0	23.0	14.7	27.9	33.3	25.3
Stage 3 (%)	36.5	39.3	43.5	25.6	66.7	39.4
Stage 4 (%)	4.8	6.6	2.9	14.0	0.0	5.3
Stage N/A (%)	4.8	14.8	11.8	16.3	0.0	11.5
Head and neck (%)	9.5	3.3	6.5	11.6	33.3	7.4
Trunk (%)	42.9	37.7	35.3	27.9	33.3	36.2
Extremities (%)	41.3	42.6	42.4	44.2	0.0	42.1
N/A and other (%)	6.3	16.4	15.9	16.3	33.3	14.4

^1^ Regional cutaneous or subcutaneous tissue, includes satellite and in-transit metastasis.

## Data Availability

The MF exome sequencing data are available on the dbGaP under accession number phs001877.v1.p1.

## References

[1] Cerroni L. (2018). Mycosis Fungoides-Clinical and Histopathologic Features, Differential Diagnosis, and Treatment. Semin. Cutan. Med. Surg..

[2] Campbell J.J., Clark R.A., Watanabe R., Kupper T.S. (2010). Sezary Syndrome and Mycosis Fungoides Arise from Distinct T-Cell Subsets: A Biologic Rationale for Their Distinct Clinical Behaviors. Blood.

[3] Iyer A., Hennessey D., O’Keefe S., Patterson J., Wang W., Wong G.K.-S., Gniadecki R. (2019). Skin Colonization by Circulating Neoplastic Clones in Cutaneous T-Cell Lymphoma. Blood.

[4] Iyer A., Hennessey D., Gniadecki R. (2022). Clonotype Pattern in T-Cell Lymphomas Map the Cell of Origin to Immature Lymphoid Precursors. Blood Adv..

[5] Ziegler A., Jonason A.S., Leffell D.J., Simon J.A., Sharma H.W., Kimmelman J., Remington L., Jacks T., Brash D.E. (1994). Sunburn and p53 in the Onset of Skin Cancer. Nature.

[6] Brash D.E. (2015). UV Signature Mutations. Photochem. Photobiol..

[7] Dousset L., Poizeau F., Robert C., Mansard S., Mortier L., Caumont C., Routier É., Dupuy A., Rouanet J., Battistella M. (2021). Positive Association Between Location of Melanoma, Ultraviolet Signature, Tumor Mutational Burden, and Response to Anti-PD-1 Therapy. JCO Precis. Oncol..

[8] Hayward N.K., Wilmott J.S., Waddell N., Johansson P.A., Field M.A., Nones K., Patch A.-M., Kakavand H., Alexandrov L.B., Burke H. (2017). Whole-Genome Landscapes of Major Melanoma Subtypes. Nature.

[9] Adami J., Frisch M., Yuen J., Glimelius B., Melbye M. (1995). Evidence of an Association between Non-Hodgkin’s Lymphoma and Skin Cancer. BMJ.

[10] Morales-Suárez-Varela M.M., Olsen J., Johansen P., Kaerlev L., Guénel P., Arveux P., Wingren G., Hardell L., Ahrens W., Stang A. (2006). Occupational Sun Exposure and Mycosis Fungoides: A European Multicenter Case-Control Study. J. Occup. Environ. Med..

[11] DeStefano C.B., Desale S., Fernandez S.J., Shenoy A.G. (2019). The Impact of Environmental Ultraviolet Exposure on the Clinical Course of Mycosis Fungoides. J. Am. Acad. Dermatol..

[12] Newton R. (1997). Solar Ultraviolet Radiation Is Not a Major Cause of Primary Cutaneous Non-Hodgkin’s Lymphoma. BMJ.

[13] McGregor J.M., Crook T., Fraser-Andrews E.A., Rozycka M., Crossland S., Brooks L., Whittaker S.J. (1999). Spectrum of p53 Gene Mutations Suggests a Possible Role for Ultraviolet Radiation in the Pathogenesis of Advanced Cutaneous Lymphomas. J. Investig. Dermatol..

[14] Jones C.L., Degasperi A., Grandi V., Amarante T.D., Mitchell T.J., Nik-Zainal S., Whittaker S.J., Genomics England Research Consortium (2021). Spectrum of Mutational Signatures in T-Cell Lymphoma Reveals a Key Role for UV Radiation in Cutaneous T-Cell Lymphoma. Sci. Rep..

[15] Wooler G., Melchior L., Ralfkiaer E., Rahbek Gjerdrum L.M., Gniadecki R. (2016). Gene Status Affects Survival in Advanced Mycosis Fungoides. Front. Med..

[16] Alexandrov L.B., Nik-Zainal S., Wedge D.C., Aparicio S.A.J.R., Behjati S., Biankin A.V., Bignell G.R., Bolli N., Borg A., Børresen-Dale A.-L. (2013). Signatures of Mutational Processes in Human Cancer. Nature.

[17] (2020). ICGC/TCGA Pan-Cancer Analysis of Whole Genomes Consortium Pan-Cancer Analysis of Whole Genomes. Nature.

[18] Iyer A., Hennessey D., O’Keefe S., Patterson J., Wang W., Salopek T., Wong G.K.-S., Gniadecki R. (2019). Clonotypic Heterogeneity in Cutaneous T-Cell Lymphoma (mycosis Fungoides) Revealed by Comprehensive Whole-Exome Sequencing. Blood Adv..

[19] SignatureAnalyzer. https://github.com/broadinstitute/SignatureAnalyzer-GPU.

[20] Pfeifer G.P., You Y.-H., Besaratinia A. (2005). Mutations Induced by Ultraviolet Light. Mutat. Res..

[21] Iyer A., Hennessey D., O’Keefe S., Patterson J., Wang W., Wong G.K.-S., Gniadecki R. (2020). Branched Evolution and Genomic Intratumor Heterogeneity in the Pathogenesis of Cutaneous T-Cell Lymphoma. Blood Adv..

[22] Sivanand A., Hennessey D., Iyer A., O’Keefe S., Surmanowicz P., Vaid G., Xiao Z., Gniadecki R. (2020). The Neoantigen Landscape of Mycosis Fungoides. Front. Immunol..

[23] Xiao M.Z.X., Hennessey D., Iyer A., O’Keefe S., Zhang F., Sivanand A., Gniadecki R. (2021). Transcriptomic Changes during Stage Progression of Mycosis Fungoides. Br. J. Dermatol..

[24] Yin Q., Shi X., Lan S., Jin H., Wu D. (2021). Effect of Melanoma Stem Cells on Melanoma Metastasis. Oncol. Lett..

[25] Schatton T., Frank M.H. (2008). Cancer Stem Cells and Human Malignant Melanoma. Pigment. Cell Melanoma Res..

[26] Schatton T., Murphy G.F., Frank N.Y., Yamaura K., Waaga-Gasser A.M., Gasser M., Zhan Q., Jordan S., Duncan L.M., Weishaupt C. (2008). Identification of Cells Initiating Human Melanomas. Nature.

[27] Peterson S.C., Eberl M., Vagnozzi A.N., Belkadi A., Veniaminova N.A., Verhaegen M.E., Bichakjian C.K., Ward N.L., Dlugosz A.A., Wong S.Y. (2015). Basal Cell Carcinoma Preferentially Arises from Stem Cells within Hair Follicle and Mechanosensory Niches. Cell Stem Cell.

[28] Grachtchouk M., Pero J., Yang S.H., Ermilov A.N., Michael L.E., Wang A., Wilbert D., Patel R.M., Ferris J., Diener J. (2011). Basal Cell Carcinomas in Mice Arise from Hair Follicle Stem Cells and Multiple Epithelial Progenitor Populations. J. Clin. Investig..

[29] Jian Z., Strait A., Jimeno A., Wang X.-J. (2017). Cancer Stem Cells in Squamous Cell Carcinoma. J. Investig. Dermatol..

[30] Bonilla X., Parmentier L., King B., Bezrukov F., Kaya G., Zoete V., Seplyarskiy V.B., Sharpe H.J., McKee T., Letourneau A. (2016). Genomic Analysis Identifies New Drivers and Progression Pathways in Skin Basal Cell Carcinoma. Nat. Genet..

[31] Martincorena I., Roshan A., Gerstung M., Ellis P., Van Loo P., McLaren S., Wedge D.C., Fullam A., Alexandrov L.B., Tubio J.M. (2015). Tumor Evolution. High Burden and Pervasive Positive Selection of Somatic Mutations in Normal Human Skin. Science.

[32] Royer-Bertrand B., Torsello M., Rimoldi D., El Zaoui I., Cisarova K., Pescini-Gobert R., Raynaud F., Zografos L., Schalenbourg A., Speiser D. (2016). Comprehensive Genetic Landscape of Uveal Melanoma by Whole-Genome Sequencing. Am. J. Hum. Genet..

[33] Wong K., Van der Weyden L., Schott C.R., Foote A., Constantino-Casas F., Smith S., Dobson J.M., Murchison E.P., Wu H., Yeh I. (2019). Cross-Species Genomic Landscape Comparison of Human Mucosal Melanoma with Canine Oral and Equine Melanoma. Nat. Commun..

[34] Yang C., Sanchez-Vega F., Chang J.C., Chatila W.K., Shoushtari A.N., Ladanyi M., Travis W.D., Busam K.J., Rekhtman N. (2020). Lung-Only Melanoma: UV Mutational Signature Supports Origin from Occult Cutaneous Primaries and Argues against the Concept of Primary Pulmonary Melanoma. Mod. Pathol..

[35] Clark R.A. (2015). Resident Memory T Cells in Human Health and Disease. Sci. Transl. Med..

[36] Clark R.A., Watanabe R., Teague J.E., Schlapbach C., Tawa M.C., Adams N., Dorosario A.A., Chaney K.S., Cutler C.S., Leboeuf N.R. (2012). Skin Effector Memory T Cells Do Not Recirculate and Provide Immune Protection in Alemtuzumab-Treated CTCL Patients. Sci. Transl. Med..

[37] Wulf H.C., Sandby-Møller J., Kobayasi T., Gniadecki R. (2004). Skin Aging and Natural Photoprotection. Micron.

[38] Gniadecka M., Wulf H.C., Mortensen N.N., Poulsen T. (1996). Photoprotection in Vitiligo and Normal Skin. A Quantitative Assessment of the Role of Stratum Corneum, Viable Epidermis and Pigmentation. Acta Derm. Venereol..

[39] Lindberg M., Boström M., Elliott K., Larsson E. (2019). Intragenomic Variability and Extended Sequence Patterns in the Mutational Signature of Ultraviolet Light. Proc. Natl. Acad. Sci. USA.

